# Impact of Right Atrial Physiology on Heart Failure and Adverse Events after Myocardial Infarction

**DOI:** 10.3390/jcm9010210

**Published:** 2020-01-12

**Authors:** Andreas Schuster, Sören J. Backhaus, Thomas Stiermaier, Jenny-Lou Navarra, Johannes Uhlig, Karl-Philipp Rommel, Alexander Koschalka, Johannes T. Kowallick, Boris Bigalke, Shelby Kutty, Matthias Gutberlet, Gerd Hasenfuß, Holger Thiele, Ingo Eitel

**Affiliations:** 1 Department of Cardiology and Pneumology, University Medical Center Göttingen, Georg-August University, German Center for Cardiovascular Research (DZHK), 37075 Göttingen, Germany; soeren.backhaus@outlook.de (S.J.B.); jenny-lou.navarra@gmx.de (J.-L.N.); alex.koscha@gmx.de (A.K.); hasenfus@med.uni-goettingen.de (G.H.); 2 Department of Cardiology, Royal North Shore Hospital, The Kolling Institute, Northern Clinical School, University of Sydney, Sydney 2065, Australia; 3 University Heart Center Lübeck, Medical Clinic II (Cardiology/Angiology/Intensive Care Medicine), University Hospital Schleswig-Holstein, German Center for Cardiovascular Research (DZHK), 23538 Lübeck, Germany; Thomas.Stiermaier@uksh.de (T.S.); ingoeitel@gmx.de (I.E.); 4 University Medical Center Göttingen, Institute for Diagnostic and Interventional Radiology, Georg-August University, German Center for Cardiovascular Research (DZHK), 37075 Göttingen, Germany; johannes.uhlig@med.uni-goettingen.de (J.U.); johannes.kowallick@med.uni-goettingen.de (J.T.K.); 5 Department of Internal Medicine/Cardiology, Heart Center Leipzig at University of Leipzig, Leipzig Heart Institute, 04289 Leipzig, Germany; Karl_Ph_Rommel@web.de (K.-P.R.); Holger.Thiele@medizin.uni-leipzig.de (H.T.); 6 Department of Cardiology and Pneumology, Charité Campus Benjamin Franklin, University Medical Center Berlin, 12203 Berlin, Germany; dr.bigalke@gmx.de; 7 Taussig Heart Center, Johns Hopkins Hospital, Baltimore, MD 21287, USA; skutty1@jhmi.edu; 8 Department of Radiology, Heart Center Leipzig, University of Leipzig, 04289 Leipzig, Germany; Matthias.Gutberlet@medizin.uni-leipzig.de

**Keywords:** myocardial infarction, risk stratification, prognosis, cardiac magnetic resonance, strain, strain rate, feature tracking, right atrium

## Abstract

Background: Right ventricular (RV) function is a known predictor of adverse events in heart failure and following acute myocardial infarction (AMI). While right atrial (RA) involvement is well characterized in pulmonary arterial hypertension, its relative contributions to adverse events following AMI especially in patients with heart failure and congestion need further evaluation. Methods: In this cardiovascular magnetic resonance (CMR)-substudy of AIDA STEMI and TATORT NSTEMI, 1235 AMI patients underwent CMR after primary percutaneous coronary intervention (PCI) in 15 centers across Germany (*n* = 795 with ST-elevation myocardial infarction and 440 with non-ST-elevation MI). Right atrial (RA) performance was evaluated using CMR myocardial feature tracking (CMR-FT) for the assessment of RA reservoir (total strain ε_s_), conduit (passive strain ε_e_), booster pump function (active strain ε_a_), and associated strain rates (SR) in a blinded core-laboratory. The primary endpoint was the occurrence of major adverse cardiac events (MACE) 12 months post AMI. Results: RA reservoir (ε_s_ *p* = 0.061, SRs *p* = 0.049) and conduit functions (ε_e_ *p* = 0.006, SRe *p* = 0.030) were impaired in patients with MACE as opposed to RA booster pump (ε_a_ *p* = 0.579, SRa *p* = 0.118) and RA volume index (*p* = 0.866). RA conduit function was associated with the clinical onset of heart failure and MACE independently of RV systolic function and atrial fibrillation (AF) (multivariable analysis hazard ratio 0.95, 95% confidence interval 0.92 to 0.99, *p* = 0.009), while RV systolic function and AF were not independent prognosticators. Furthermore, RA conduit strain identified low- and high-risk groups within patients with reduced RV systolic function (*p* = 0.019 on log rank testing). Conclusions: RA impairment is a distinct feature and independent risk factor in patients following AMI and can be easily assessed using CMR-FT-derived quantification of RA strain.

## 1. Introduction

Cardiovascular disease, and especially myocardial infarction, are of significant clinical importance [1,2]. Left ventricular ejection fraction (LVEF) is most commonly used for risk-stratification, but does not provide information on regional cardiac function [3,4]. Recently, incremental value for LV myocardial deformation assessments has been demonstrated [5,6]. Myocardial strain allows precise quantification of global and regional cardiac function, and is consequently used as an endpoint in many pharmacological studies [7,8]. Beyond LV functional analyses, associations of left atrial involvement and cardiovascular diseases [9], as well as mortality [10], have been established following acute myocardial infarction (AMI) [11] and in heart failure with both preserved and reduced LVEF [12].

Cardiovascular magnetic resonance (CMR) represents the reference standard for cardiac morphology and function assessment [13,14], including the right atrium [15]. CMR feature tracking (CMR-FT) allows the assessment of myocardial deformation based on routinely acquired balanced steady-state free precession (bSSFP) cine images. The feasibility of atrial strain assessment has been shown for both the left (LA) [16] and right (RA) [17] atrium, and may be incremental to simplistic volumetric approaches [18,19]. 

In contrast to evidence indicating a great role of LV [5,20] and LA [11,21] physiology following AMI, evidence for the impact of RA function is scarce. RA strain is associated with right atrial pressure [22,23] and with functional capacity in pulmonary arterial hypertension [22], potentially serving as a compensatory feature of impaired RV stroke volume [24]. Such compensatory impact on LV- and global cardiac function following AMI is likely but has never been adequately studied in a prospective multicenter setting. Preliminary evidence suggests a potential role for RA function in chronic heart failure [25] and in patients with coronary artery disease [15]. Consequently, the aim of the present study was the characterization of RA involvement in the clinical course following AMI. 

## 2. Methods

### 2.1. Study Population

This CMR substudy includes the patients from two previously published infarction cohorts who underwent post interventional additional CMR scanning during their hospitalization. Firstly, the STEMI cohort taken from the AIDA STEMI (Abciximab Intracoronary versus intravenously Drug Application in STEMI) trial registered on ClinicalTrials.gov under NCT00712101 [26], and secondly, the NSTEMI cohort taken from the TATORT-NSTEMI (Thrombus Aspiration in Thrombus Containing Culprit Lesions in Non–ST-Elevation) trial registered with ClinicalTrials.gov under NCT01612312 [27].

The open label AIDA STEMI study enrolled 2065 patients in 22 study sites across Germany who were then randomized, comparing intracoronary (*n* = 1.032) to intravenous (*n* = 1.033) abciximab bolus application (0.25 mg/kg bodyweight) during primary percutaneous coronary intervention (PCI). Of these, 795 patients underwent additional CMR scanning at eight of these sites, which were chosen due to their expertise. 

The TATORT NSTEMI trial was conducted prospectively, enrolling 440 patients at seven study sites across Germany. Patients were randomized for either aspiration thrombectomy (*n* = 221) or standard PCI (*n* = 219), investigating the effects on microvascular injury assessed by CMR. Neither AIDA STEMI nor TATORT NSTEMI showed differences in their treatment arms. The studies were approved by both the lead ethical committee located at the University of Leipzig as well as all local ethical committees of involved centers. All patients gave written informed consent before randomization. The studies were conducted according to the principles of the Helsinki Declaration. The predefined CMR substudy was supported by a grant of the German Centre of Cardiovascular Research (DZHK).

### 2.2. Cardiovascular Magnetic Resonance Imaging Protocol and Analyses

All individual study sites employed one identical CMR imaging protocol on clinical 1.5 or 3.0 Tesla MR scanners [28]. CMR was conducted within the first ten days after the index event acquiring long axis 2- and 4-chamber views (CV), as well as the short axis (SA) stack. Exclusion criteria met the typical contraindications for CMR [3,28]. Myocardial infarction was characterized using LVEF, microvascular obstruction (MO), myocardial salvage, and infarct size (IS) [3,28]. RA was assessed by RA volume index (RAVI, maximal RA volume/m²), as well as CMR-FT based strain analyses [17]. The dedicated feature tracking software offers a reproducible [17,29,30,31], fast, and detailed assessment of atrial strain [16,17] in routinely-acquired b-SSFP cine images.

**Figure 1:**
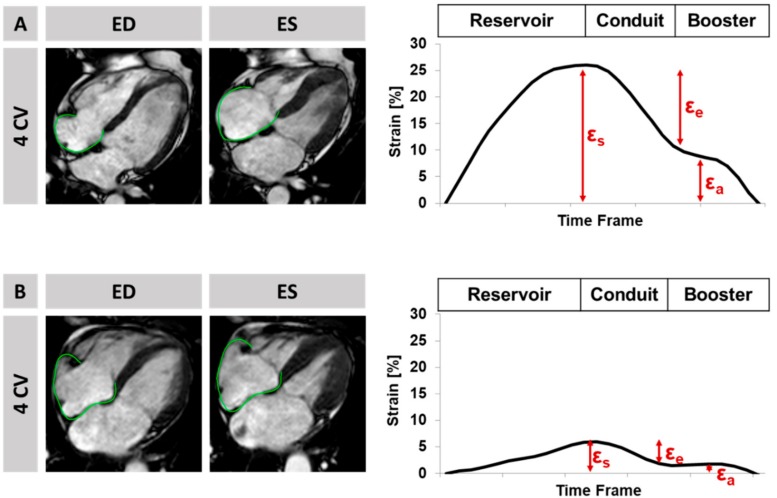
Feature-Tracking and Strain analysis on the left, display of a four chamber view (CV) with endocardially-tracked borders in the right atrium shown at end-diastole and systole, on the right, display of the corresponding curves of reservoir (ε_s_), conduit (ε_e_), and booster pump (ε_a_) for a patient with (**A**) normal left ventricular ejection fraction of 56% and no major adverse cardiac event during follow-up, and (**B**) normal left ventricular ejection fraction of 52% and a major adverse clinical event during follow-up.

Briefly, strain analyses were performed by fully blinded operators on b-SSFP images using dedicated validated and established offline postprocessing software (2D CPA MR, Cardiac Performance Analysis, Version 1.1.2, TomTec Imaging Systems, Unterschleissheim, Germany) in an experienced core laboratory with excellent intra- and inter- observer reproducibility at the University Medical Center Goettingen [16,29,30,32]. First, end-diastolic RA endocardial borders were manually traced. The tracking algorithm was applied following 48 features over the cardiac cycle. Tracking accuracy was visually reviewed; where needed, corrections were made to the initial contour only. The results are based on the average of three independently repeated measurements in the 4 CV [31]. Atrial strain analyses assess the three physiological functions of atrial mechanics, i.e., the first RA reservoir function (total strain ε_s_) representing the collection of venous return during ventricular systole, the conduit function (passive strain ε_e_) for the passive early diastolic blood passage during ventricular filling, and finally, the booster pump function (active strain ε_a_) for the active late diastolic augmentation of ventricular filling (Figure 1) [16].

### 2.3. Clinical Endpoints

The primary clinical endpoint of this study was the occurrence of major adverse cardiovascular events (MACE) including all-cause death, reinfarction, or congestive heart failure during the first year after the initial event. To avoid statistical falsification, each patient could only account for one event according to the respective severity (death > reinfarction > congestive heart failure). A blinded clinical endpoints committee adjudicated all components of the combined clinical endpoints on the basis of data provided by the clinical trial sites. Detailed patient outcome and definitions have been published previously [26,27,33].

### 2.4. Statistics

Statistical calculations were performed using IBM SPSS Statistic Software Version 24 for Windows (IBM, Armonk, NY, USA), R version 3.3.2 (R Core Development Team, Vienna, Austria) and RStudio version 1.0.44 (RStudio Inc., Boston, MA, USA). All *p*-values provided are two-sided; an alpha level of 0.05 and below was considered statistically significant. Categorical parameters are reported in absolute numbers and related percentage values. Continuous parameters are presented as median with interquartile range (IQR). Differences between categorical variables were compared using the chi-square test. Independent continuous parameters were compared using the Mann-Whitney-U test. Continuous parameters were checked for normal distribution based on the Shapiro-Wilk test. The correlation of non-normally distributed parameters was evaluated using the Spearman’s rank correlation coefficient. Clinical endpoint analyses were performed using Kaplan-Meier plots and associated log-rank tests. Linear univariate Cox regression was used for calculation of hazard ratios (HR) which are given with corresponding 95% confidence intervals (CI). Multivariable Cox regression models were then performed for further statistical adjustments of univariate significant factors. Reproducibility was tested within 30 (15 STEMI, 15 NSTEMI) randomly selected patients. To assess intraobserver variability, these cases were analyzed twice, with at least four weeks in between repeated analyses by a blinded operator. For interobserver variability, these cases were analyzed by a second blinded operator. Calculations comprised Bland-Altman analysis [34], intraclass correlation coefficients (ICC), and coefficients of variation (CoV, standard deviation of the differences divided by the mean). Agreements are considered excellent ICC > 0.74, good 0.60–0.74, fair 0.4–0.59, or poor < 0.4 [30].

## 3. Results

### 3.1. Study Population

**Figure 2:**
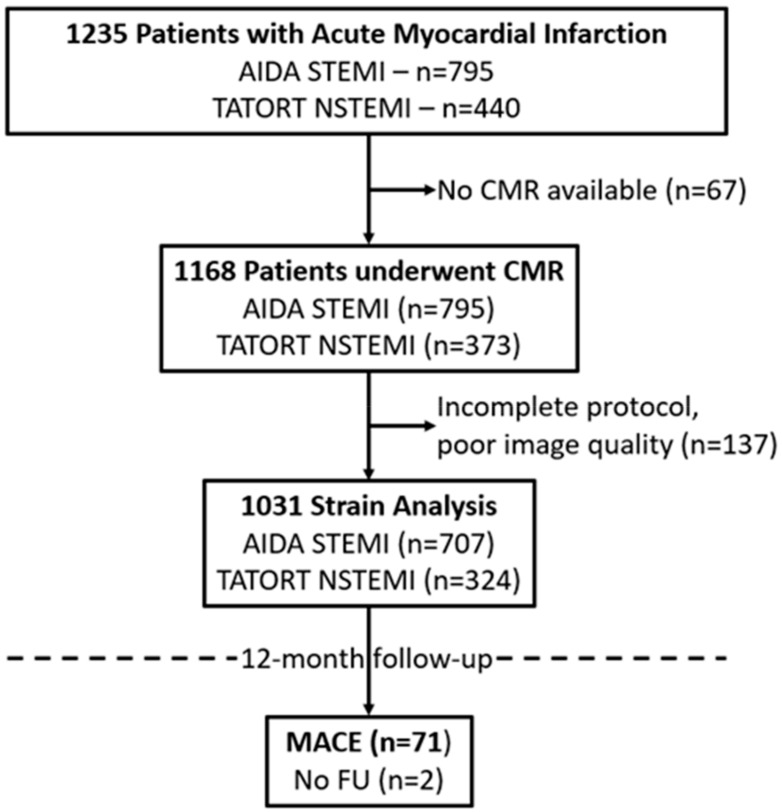
Study Flow-Chart. CMR = cardiovascular magnetic resonance, MACE = major adverse cardiovascular events, N/STEMI = non/ ST-elevation myocardial infarction.

The study population, with a median age of 64 years (53, 72), consisted of 1235 patients, of which *n* = 795 were recruited in AIDA STEMI and *n* = 440 in TATORT NSTEMI. CMR, performed in median on day three (IQR 2–4) after the index event, was available in 1031 patients with regards to complete imaging protocols and image quality for FT postprocessing (Figure 2). Follow-up was achieved in 99.8% of all patients with available FT data. Of these patients, 71 had a predefined event within the first year after infarction (death *n* = 29, reinfarction *n* = 25 and/or readmission due to congestive heart failure *n* = 35). 

Baseline characteristics and comparisons between MACE and no MACE are reported in Table 1. Patients with predefined MACE were significantly older (*p* < 0.001), suffered more frequently from hypertension (*p* = 0.019), and smoking was less common amongst them (*p* = 0.039). Killip class on admission (*p* < 0.001) and the number of diseased vessels (*p* = 0.025) were significantly worse in patients with MACE, whilst time to treatment, the affected artery, pre- and post- interventional TIMI flow, as well as number of implanted stents were similar. 

Overall, patients had a preserved LVEF of 50.5% (IQR 43.5%, 57.5%) and RVEF of 61.1% (IQR 54.2%, 67.7%), an IS of 13.1% LV (IQR 5.2%, 21.7%) with an area at risk of 29.1%LV (IQR 20.1%, 42.2%), a myocardial salvage index of 54.7% (IQR 34.8%, 74.9%), and a microvascular obstruction of 0.33%LV (IQR 0%, 1.9%) as assessed by CMR. Of these, LVEF (*p* < 0.001), RVEF (*p* = 0.037), IS (*p* = 0.001) and myocardial salvage index (*p* = 0.032) were significantly more impaired in patients with MACE, while MO (*p* = 0.06) and area at risk (*p* = 0.08) did not reach statistical significance.

### 3.2. Right Atrial Dysfunction

CMR-derived RA functional parameters are reported in Table 2, and RA strain reproducibility is shown in Table 3. RA conduit function showed the most significant difference between patients with and without MACE (ε_e_ *p* = 0.006, SRe *p* = 0.03), followed by reservoir function (ε_s_ *p* = 0.061, SRs *p* = 0.049). Booster pump function was similar in both groups (ε_a_ *p* = 0.579, SRa *p* = 0.118) (Table 2). Similarly, discrimination between patients admitted for rehospitalization due to heart failure was best for conduit (ε_e_ *p* = 0.003, SRe *p* = 0.008), followed by reservoir (ε_s_ *p* = 0.029, SRs *p* = 0.087) function. Booster pump strain did not provide risk discrimination (ε_a_ *p* = 0.909, SRa *p* = 0.367). Conduit (ε_e_ *p* < 0.001, SRe *p* = 0.007) but not reservoir (ε_s_ *p* = 0.068, SRs *p* = 0.709) or booster pump function (ε_a_ *p* = 0.341, SRa *p* = 0.148) discriminated between patients without (Killip class = 1) and with clinical signs of heart failure (Killip class ≥ 2). Body surface area (BSA)-indexed maximum RA volumes were not significantly different between patients with and without MACE (*p* = 0.866) (Table 2) or heart failure (*p* = 0.758). RA function was significantly decreased in patients with atrial fibrillation (AF) (ε_s_ *p* < 0.001, ε_e_ *p* < 0.001 and ε_a_ *p* = 0.022).

RA function as assessed by strain and SR parameters did not correlate with IS (*p* > 0.167 for all). Correlation between RA performance and RVEF was weak but highly significant for all atrial functional parameters (RA ε_s_ *r* = 0.19, SRs *r* = 0.23, ε_e_ *r* = 0.15, SRe *r* = −0.17, ε_a_ *r* = 0.14, and SRa −0.13, *p* < 0.001 for all). Similarly, correlation with LVEF was weak and strongly significant for reservoir and conduit function (RA ε_s_ *r* = 0.14, SRs *r* = 0.13, ε_e_ *r* = 0.19 and SRe *r* = −0.15, *p* < 0.001 for all), while booster pump function did not correlate with LVEF (ε_a_ *r* = 0.06, *p* = 0.073 and SRa −0.05, *p* = 0.104). 

### 3.3. Risk Stratification

In univariate Cox regression (Table 4), only RA conduit function had a highly statistically significant impact on MACE occurrence (HR 0.95, 95% CI 0.91 to 0.98, *p* = 0.003), while reservoir strain did not reach statistical significance (HR 0.98, 95% CI 0.96 to 1.00, *p* = 0.062). Booster pump function did not predict MACE (HR 1.00, 95% CI 0.98 to 1.04, *p* = 0.745). Similarly, RAVI did not impact outcome (HR 1.01, 95% CI 0.99–1.02, *p* = 0.634). AF was significantly associated with the incidence of MACE (HR 2.25, 95% CI 1.20–4.23, *p* = 0.011) (Table 3). Considering heart failure separately regarding outcome both conduit (ε_e_ *p* = 0.003, SRe *p* = 0.008) and reservoir functions (ε_s_ *p* = 0.029) were impaired in these patients and identified increased risk (ε_e_ HR 0.92, 95% CI 0.87 to 0.97, *p* = 0.004, ε_s_ HR 0.96, 95% CI 0.93 to 1.00, *p* = 0.027). Booster pump had no impact (HR 1.00 95% CI 0.95 to 1.04, *p* = 0.830). 

In multivariable Cox-regression analyses including RVEF, AF, and passive conduit function (Ee) using continuous parameters, passive conduit function revealed independence, irrespective of RVEF and AF for MACE (RA ε_e_ HR 0.95, 95% CI 0.92 to 0.99, *p* = 0.009), whilst RVEF (RVEF HR 0.98. 95% CI 0.96–1.00, *p* = 0.085) and AF (HR 1.59, 95% CI 0.75–3.40, *p* = 0.227) did not emerge as independent prognostic factors. A trend remained for passive conduit RA function (Ee, HR 0.97, 95% CI 0.93–1.01, *p* = 0.088) if LV EF was additionally to be included (LVEF, HR 0.94, 95% CI 0.92–0.97, *p* < 0.001). 

**Figure 3:**
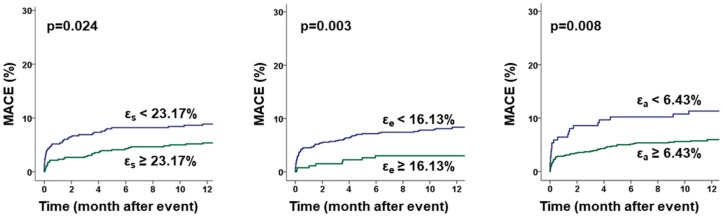
Influence of RA Strain on MACE prediction. The graphs show the influence of right atrial (RA) reservoir (ε_s_), conduit (ε_e_) and booster pump (ε_a_) strain on the rate of major adverse clinical events (MACE) during 12 months follow-up after ROC-adapted dichotomization, *p* values calculated by log-rank test.

**Figure 4:**
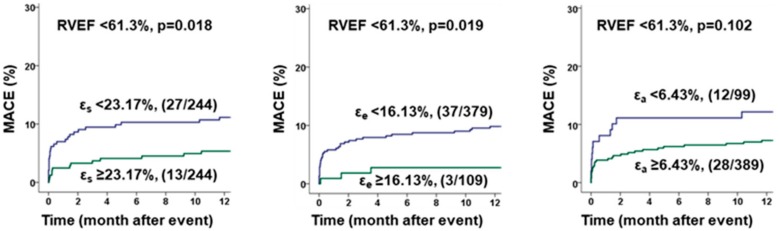
Benefit of additional right atrial strain analysis over sole right ventricular function. The graphs show the impact of right atrial (RA) reservoir (ε_s_), conduit (ε_e_) and booster pump (ε_a_) function evaluation on the occurrence of major adverse cardiac events (MACE). RA function was investigated in addition to right ventricular ejection fraction (RVEF) below the median. Cut-Offs for RA strains were chosen for optimal sensitivity and specificity, and *p* values were calculated by log-rank test for the occurrence of major adverse clinical events (MACE) within 12 months after myocardial infarction.

Kaplan-Meier Plots and associated Log-rank testing revealed a significant impact of atrial function on MACE occurrence (ε_s_ cut off 23.17%, *p* = 0.024; ε_e_ cut off 16.13%, *p* = 0.003 and ε_a_ cut off 6.43%, *p* = 0.008) after ROC optimized dichotomization (Figure 3). Additionally, atrial function discriminated elevated risks for patients with RVEF below the median (Figure 4). 

## 4. Discussion

The present study reports the value of CMR-FT derived data on RA performance following AMI. The study bears several notable findings regarding RA function and associated passive and active phases. RA passive conduit function reveals the highest association with MACE. Passive atrial restoring forces are associated with outcome independently of RV systolic function. Furthermore, they are associated with clinical onset of heart failure symptoms. Although there is a significant correlation of atrial functional phases and both right and left ventricular systolic function, correlation is low, and indicates a significant independent contribution of RA performance beyond systolic biventricular function.

Impaired conduit function showed the highest association with increased risk for adverse clinical events. A decrease of RA passive emptying fraction has been shown for patients with increased pulmonary artery pressure [35], coronary slow-flow [36], and left ventricular dysfunction related to left ventricular hypertrophy [37] and heart failure with preserved ejection fraction (HFpEF) [38]. On the one hand, RA conduit function is connected to and reflects right and left ventricular (dys) function, as described by a significant correlation of passive conduit function with systolic RV and LV function. RA conduit strain is strongly related to early RV filling in HFpEF [38], thus contributing to RV and, subsequently, LV stroke volumes. On the other hand, correlation was low indicating value of atrial function beyond systolic biventricular function. At part, impaired RA conduit function may also imply diastolic RV dysfunction. The onset of diastolic dysfunction forestalls systolic dysfunction during the ischemic cascade [39], and a link between total LA strain and LV filling pressures has previously been made [40]. Reports also indicate independence of LA passive conduit function of LV stiffness [41]; thus, RA passive restoring forces (conduit function) themselves may have a distinct role in cardiac pathophysiology and patients with AMI beyond RV function. Indeed, the value of RA conduit function grows beyond sole RV function. In HFpEF, RA conduit function showed strong associations with maximum oxygen uptake independent of sole RV stiffness and relaxation [38]. Impaired RA function was significantly associated with heart failure symptoms as assessed using Killip class and heart failure readmission. AF was associated with reduced atrial function, including all three atrial functional phases. Indeed, impaired RA strain is a known predictor of AF reoccurrence in paroxysmal AF [42]. Importantly, passive conduit function provided risk stratification for MACE independently of systolic RV function and AF, whilst RVEF and AF were not independently associated with outcome. Additionally, RA conduit function provided incremental value for risk stratification in patients presenting with RVEF below the median. However, the impact of RA function on MACE occurrence was not independent of LV function, as shown by multivariable analyses with only a trend remaining for RA Ee.

Quantification of total RA function is challenging [43]. It is noteworthy that the overall reservoir/total strain values assessed in this study are similar to previously published reference values [44], underlining consistency. Total LA strain is an independent risk factor for MACE beyond LV function [11], and is able to compensate for LV heart failure in left anterior descending lesions [45]. RA reservoir strain was significantly associated with increased risk for heart failure and after ROC-adapted dichotomization reservoir function identified elevated risk for MACE in Kaplan-Meier plots, providing incremental value for risk stratification in patients with RVEF below the median. On the one hand side, impaired RA function leads to impaired systolic RV function and, subsequently, LV preload and cardiac output [46]. However, in contrast to passive atrial function reflecting early RV filling and elastic atrial restoring forces, RA reservoir function is associated with pulmonary artery pressures caused by LV heart failure [47], and may rather reflect the degree of congestion. Since BSA-indexed RA volumes were similar comparing patients with and without MACE, this fact could explain why passive atrial strain is distinctly more precise in the identification of patients at risk compared to total atrial strain.

## 5. Limitations

Several limitations need to be addressed for accurate interpretation of the findings. First, this CMR substudy included patients from the multicenter AIDA STEMI and TATORT NSTEMI trials; thus, CMR was performed in eight centers using different CMR vendors and sequences. However, the study sites were chosen for their expertise and employed the same imaging protocol. Furthermore, data analyses were performed in one experienced core-laboratory. Second, due to the acute nature of AMI and diverse impacts on each patient, the optimal time-point of CMR may vary. While with a median of three (IQR 2–4) days a consistent time point was achieved, little is known about the optimal time-point for CMR and its impact on risk stratification. Additionally, sicker patients potentially underwent CMR later or not at all, resulting in a possible selection bias. However, a large number of patients, i.e., 1031, post PCI has been prospectively enrolled shortly after intervention, compared to only 67 patients who did not undergo CMR imaging. Lastly, data on estimated systolic pulmonary artery pressure, which may influence RA function, was not systematically assessed, and is consequently not available.

## 6. Conclusions

RA function is associated with onset of clinical heart failure symptoms and outcome. Amongst the three atrial functional phases, RA passive conduit function most precisely identifies the degree of heart failure, as well as patients at risk for MACE. RA conduit function adds incremental prognostic value beyond the assessment of systolic ventricular function following AMI, suggesting an important contribution to global cardiac mechanics. 

## Figures and Tables

**Table 1:** Baseline characteristics.

Variable	All Patients *n* = 1031	MACE *n* = 71	No MACE *n* = 960	*p*-Value
Age	64 (53, 72)	71 (60, 77)	63 (52, 72)	**<0.001**
Sex (m)	774/1029 (75.2%)	47/71 (66.2%)	727/958 (75.9%)	0.068
**Cardiovascular risk factors**				
Active smoking	415/951 (43.6%)	20/64 (31.3%)	395/887 (44.6%)	**0.039**
Hypertension	728/1027 (70.6%)	59/71 (83.1%)	669/956 (70%)	**0.019**
Hyperlipoproteinemia	387/1024 (37.8%)	25/71 (35.2%)	362/953 (38%)	0.642
Diabetes	243/1027 (23.7%)	23/71 (32.4)	220/956 (23%)	0.073
Body mass index (kg/m²)	27.5 (24.9, 30.4)	27.7 (25.5, 31.1)	27.5 (24.9, 30.3)	0.433
Previous myocardial infarction	72/1027 (7%)	4/71 (5.6%)	68/956 (7.1%)	0.638
Previous PCI	89/1028 (8.7%)	5/71 (7%)	84/957 (8.8%)	0.616
Previous CABG	19/1028 (1.8%)	2/69 (2.9%)	17/957 (1.8%)	0.530
ST-segment elevation	707/1029 (68.7%)	48/71 (67.6%)	659/958 (68.8%)	0.836
Systolic blood pressure (mmHg)	133 (119, 150)	130 (110, 150)	134 (120, 150)	0.166
Diastolic blood pressure (mmHg)	80 (70, 89)	77 (65, 85)	80 (70, 89)	0.059
Heart rate (beats/min)	76 (67, 86)	80 (70, 96)	76 (66, 86)	**0.001**
Time symptoms to balloon * (min)	180 (110, 310)	194 (114, 390)	180 (109, 306)	0.279
Door-to-balloon time * (min)	30 (22, 42)	28 (22.5, 40)	30 (22, 42)	0.490
**Killip class on admission**				**<0.001**
1	911/1029 (88.7%)	46/71 (64.8%)	865/958 (90.3%)	
2	81/1029 (7.9%)	16/71 (22.5%)	65/958 (6.8%)	
3	22/1029 (2.1%)	5/71 (7%)	17/958 (1.8%)	
4	15/1029 (1.5%)	4/71 (5.6%)	11/958 (1.1%)	
**Diseased vessels**				**0.025**
1	517/1029 (50.3%)	27/71 (38%)	490/958 (51.1%)	
2	311/1029 (30.3%)	22/71 (31%)	289/958 (30.2%)	
3	201/1029 (19.6%)	22/71 (31%)	179/958 (18.7%)	
**Affected artery**				0.337
left anterior descending	425/1029 (41.4%)	37/71 (52.1%)	388/958 (40.5%)	
left circumflex	213/1029 (20.7%)	14/71 (19.7%)	199/958 (20.8%)	
left main	4/1029 (0.4%)	0/71 (0%)	4/958 (0.4%)	
right coronary artery	381/1029 (37.1%)	20/71 (28.2%)	361/958 (37.7%)	
bypass graft	6/1029 (0.6%)	0/71 (0%)	6/958 (0.6%)	
**TIMI flow grade before PCI**				0.607
0	515/1029 (50.1%)	40/71 (56.3%)	475/958 (49.6%)	
1	111/1029 (10.8%)	5/71 (7%)	106/958 (11.1%)	
2	215/1029 (20.9%)	13/71 (18.3%)	202/958 (21.1%)	
3	188/1029 (18.3%)	13/71 (18.3%)	175/958 (18.3%)	
Stent implanted	1005/1029 (97.9%)	69/71 (97.2%)	936/958 (97.7%)	0.524
**TIMI flow grade after PCI**				0.294
0	20/1029 (1.9%)	1/71 (1.4%)	19/958 (2%)	
1	21/1029 (2.0%)	3/71 (4.2%)	18/958 (1.9%)	
2	76/1029 (7.4%)	8/71 (11.3%)	68/958 (7.1%)	
3	912/1029 (88.8%)	59/71 (83.1%)	853/958 (89%)	
**Medication**				
Glycoprotein IIb/IIIa inhibitor	729/1028 (70.9%)	51/71 (71.8%)	678/957 (70.8%)	0.860
Aspirin	1027/1029 (99.8%)	71/71 (100%)	956/958 (99.8%)	0.700
Clopidogrel/Prasugrel/Ticagrelor	1028/1028 (100%)	71/71 (100%)	957/957 (100%)	
Betablocker	983/1028 (95.6%)	69/71 (97.2%)	914/957 (95.5%)	0.505
ACE-inhibitor/AT-1 antagonist	947/1028 (92.1%)	67/71 (94.4%)	880/957 (92%)	0.467
Aldosterone antagonist	133/1028 (12.9%)	23/71 (32.4%)	110/957 (11.5%)	**<0.001**
Statin	990/1028 (96.3%)	69/71 (97.2%)	921/957 (96.2%)	0.684
Time to MRI (days)	3 (2, 4)	3 (2, 4)	3 (2, 4)	0.06

Data presented as n/N (%) or median (IQR). *p*-values were calculated for the comparison between patients with and without MACE. Numbers in bold type indicate a significant difference. * only assessed in STEMI patients (*n* = 795), CABG = coronary artery bypass graft; MACE = major adverse cardiac event; PCI = percutaneous coronary intervention; TIMI = Thrombolysis In Myocardial Infarction. *p*-values in bold indicate statistical significance < 0.05.

**Table 2:** Atrial Performance.

	All Patients	MACE	No MACE	
**Functional parameter**	median (IQR)	median (IQR)	median (IQR)	*p*
**Infarct characteristics**				
IS	13.1 (5.20, 21.7)	20.3 (9.83, 28.9)	12.8 (5.15, 21.3)	**0.001**
AAR	29.1 (20.1, 42.2)	32.9 (24.2, 45.1)	28.8 (20.0, 42.0)	0.080
MO	0.33 (0.00, 1.92)	0.80 (0.00, 2.53)	0.29 (0.00, 1.90)	0.060
**Left ventricle**				
LV mass	66.1 (57.4, 75.9)	68.9 (58.9, 78.7)	65.9 (57.2, 75.8)	0.380
EDV	73.3 (62.5, 86.0)	75.4 (67.0, 87.5)	73.1 (62.1, 85.8)	0.155
ESV	35.6 (27.8, 45.9)	45.1 (31.6, 54.1)	35.2 (27.6, 45.3)	**<0.001**
EF	50.5 (43.5, 57.5)	41.2 (33.0, 52.2)	51.0 (44.5, 57.6)	**<0.001**
Strain	−16.6 (−12.5, −20.2)	−11.7 (−8.18, −17.1)	−16.8 (−13.0, −20.4)	**<0.001**
**Left atrium**				
LAVI	35.0 (26.6, 44.3)	40.6 (28.7, 53.6)	34.6 (26.6, 43.4)	**0.001**
LA Es	20.9 (16.2, 25.7)	16.2 (11.4, 21.1)	21.2 (16.7, 26.1)	**<0.001**
LA Ee	8.69 (5.63, 11.7)	6.92 (3.19, 8.73)	8.83 (5.83, 11.9)	**<0.001**
LA Ea	11.5 (8.60, 15.3)	9.96 (5.91, 12.7)	11.7 (8.77, 15.5)	**<0.001**
LA SRs	0.88 (0.70, 1.08)	0.79 (0.59, 0.93)	0.90 (0.71, 1.10)	**<0.001**
LA SRe	−0.55 (−0.38, −0.78)	−0.48 (−0.34, −0.67)	−0.56 (−0.39, −0.79)	**0.004**
LA SRa	−0,96 (−0.73, −1.25)	−0.84 (−0.59, −1.06)	−0.97 (−0.73, −1.26)	**0.001**
**RV volumes**				
RV mass	22.2 (18.9, 26.2)	20.8 (19.4, 24.9)	22.2 (18.9, 26.4)	0.247
EDV	60.9 (51.3, 71.4)	59.8 (48.0, 68.2)	61.0 (51.6, 71.5)	0.122
ESV	23.1 (17.4, 31.2)	23.1 (16.3, 35.6)	23.1 (17.5, 30.8)	0.878
EF	61.1 (54.2, 67.7)	56.5 (46.1, 69.4)	61.3 (54.6, 67.7)	**0.037**
**Right atrium**				
RAVI	27.4 (20.7, 35.7)	26.7 (18.7, 36.3)	27.4 (20.8, 35.6)	0.866
RA Es	24.4 (17.3, 32.4)	22.1 (13.9, 30.7)	24.8 (17.5, 32.5)	0.061
RA Ee	10.9 (6.03, 16.5)	8.88 (3.99, 13.9)	11.1 (6.18, 16.7)	**0.006**
RA Ea	12.3 (7.89, 17.5)	11.4 (6.31, 18.6)	12.3 (8, 17.4)	0.579
RA SRs	1.11 (0.83, 1.43)	0.98 (0.64, 1.43)	1.11 (0.84, 1.43)	**0.049**
RA SRe	−0.54 (−0.33, −0.79)	−0.48 (−0.21, -0.65)	−0.55 (−0.34, −0.8)	**0.030**
RA SRa	−0.96 (−0.66, −1.37)	−0.89 (−0.52, −1.28)	−0.97 (−0.67, −1.37)	0.118

IQR = interquartile range, IS = infarct size, AAR = area at risk, MO = microvascular obstruction, RA/V = right atrium/ventricle, Es/SRs = reservoir strain/rate, Ee/SRe = conduit strain/rate and Ea/SRa = booster pump strain/rate, LAVI/RAVI = left/right atrial volume index. *p*-values in bold indicate statistical significance < 0.05.

**Table 3:** Reproducibility of right atrial strain assessment.

	Mean Difference ± SD (%)	CoV (%)	ICC (95% CI)
**Intraobserver**			
RA Es	−2.05 ± 3.84	13.73	0.95 (0.86–0.98)
RA Ee	−0.17 ± 2.23	16.95	0.97 (0.94–0.99)
RA Ea	−1.98 ± 4.58	30.84	0.83 (0.62–0.92)
RA SRs	−0.08 ± 0.32	26.02	0.82 (0.63–0.92)
RA SRe	0.02 ± 0.17	31.48	0.91 (0.80–0.96)
RA SRa	0.08 ± 0.29	25.66	0.85 (0.69–0.93)
**Interobserver**			
RA Es	1.67 ± 6.23	23.86	0.87 (0.72–0.94)
RA Ee	1.22 ± 4.14	33.15	0.88 (0.74–0.94)
RA Ea	0.44 ± 3.44	25.26	0.93 (0.84–0.97)
RA SRs	0.01 ± 0.29	25.44	0.84 (0.66–0.93)
RA SRe	0.02 ± 0.20	37.04	0.88 (0.74–0.94)
RA SRa	−0.03 ± 0.29	27.10	0.86 (0.70–0.93)

SD = standard deviation, CoV = coefficient of variation, ICC = intraclass correlation coefficient, RA Es/e/a and SRs/e/a = right atrial reservoir, conduit and booster pump strain, and associated strain rate.

**Table 4:** Predictors of MACE in univariable Cox regression analysis.

Variable	Univariable Hazard Ratio (CI)	*p*
Age	1.04 (1.02–1.06)	**<0.001**
Hypertension	2.07 (1.11–3.84)	**0.022**
LVEF	0.94 (0.92–0.96)	**<0.001**
RVEF	0.97 (0.95–0.99)	**0.012**
Infarct Size	1.03 (1.01–1.05)	**0.001**
Killip Class	2.08 (1.64–2.64)	**<0.001**
Number of diseased vessels	1.46 (1.10–1.94)	**0.009**
Atrial fibrillation	2.25 (1.20–4.23)	**0.011**
RA-Es	0.98 (0.96–1.00)	0.062
RA-Ee	0.95 (0.91–0.98)	**0.003**
RA-Ea	1.00 (0.98–1.04)	0.745
RAVI	1.01 (0.99–1.02)	0.634

CI = confidence interval, LV/RF-EF = left/right ventricular ejection fraction, RA = right atrium, Es = reservoir strain, Ee = conduit strain, Ea = booster pump strain, and RAVI = right atrial volume index. *p*-values in bold indicate statistical significance < 0.05.
